# Synthesis, Characterization and Cytotoxicity of Alkylated Quercetin Derivatives

**Published:** 2016

**Authors:** Xin-Ran Bao, Han Liao, Jiao Qu, Yong Sun, Xin Guo, En-Xia Wang, Yu-Hong Zhen

**Affiliations:** a *Clinical Medicine of Seven-year-program, Dalian Medical University, No.9 Lvshun Southern Road, Lvshunkou District, Dalian 116044, P. R. China. *; b *College of Pharmacy, Dalian Medical University, No.9 Lvshun Southern Road, Lvshunkou District, Dalian 116044, P. R. China.*

**Keywords:** Quercetin, Alkyl derivatives, Cytotoxicity, Selective alkylation

## Abstract

Quercetin, a ubiquitous flavonol, represents a promising leading drug for development of new chemotherapeutic agents. However, its limited cytotoxicity to cancer cells hampers its clinical use. In order to obtain novel quercetin derivatives with superior cytotoxicity, seven alkylated quercetin derivatives were synthesized. Solubility of these derivatives was determined by turbidimetry. Cytotoxicity of the high-soluble derivatives against MCF-7 cells and caco-2 cells was determined using MTT assay. Among these seven products, 7-*O*-butylquercetin had the highest solubility in DMEM medium and 7-*O*-geranylquercetin had the most potent cytotoxicity. Further study on cytotoxicity of 7-*O*-geranylquercetin on NCI-H446, A549, MGC-803 and SGC-7901 cell lines revealed potential antiproliferative effects. The 7-*O*-geranylquercetin is a broad spectrum cytotoxic agent and it may be a promising leading drug for cancer chemotherapy.

## Introduction

Quercetin is a mild cytotoxic agent and a potential candidate for treating many kinds of cancer (1-3). To find a possible chemotherapeutic drug for clinical use, interest in quercetin derivatives for cancer treatment has increased over the past ten years. However, the cytotoxicity of the reported quercetin derivatives is still not strong enough. A water-soluble derivative, quercetin-5′,8-disulfonate, showed increased cytotoxicity to about 1.5-fold relative to quercetin (4). Comparing with quercetin, 3,7,3′,4′-*O*-tetraacetylquercetin increases the cytotoxicity about 3-fold while 3,7,3*′*,4*′*-*O*-tetramethylquercetin decreases the cytotoxicity (5). This shows that non-specific *O*-acetylation, *O*-alkylation and sulfonation are not efficient ways for increasing the cytoxicity of quercetin.

A previous study indicated that the cytotoxicity of quercetin is related to its oxidative products, and only 7-*O*-semiquinone is harmless to cells (6). Consequently, 7-*O*alkylated derivatives might have higher cytotoxicity because these derivatives could not form the harmless 7-*O*-semiquinone.

To obtain novel derivatives with high cytotoxicity, seven alkyl derivatives of quercetin were synthesized, purified and characterized in this study. The derivatives with high solubility were further studied to determine their cytotoxic effects on different cancer cell lines. 

## Experimental

*Materials*

All reagents and solvents used are commercially available. All the cell lines were purchased from ATCC and cultured as described in ATCC. UV spectra were determined on a JASCO V-650 spectrophotometer, and ^1^H NMR, ^13^CNMR and NOESY spectra were recorded on a Bruker AVANCE 400-MHz instrument in DMSO-*d6* (Bruker Corporation, Zurich, Switzerland). The molecular weights were measured on HP 1100LC/MSD spectrometer (Agilent Technologies Corporation, Santa Clara, CA, USA). 

*General synthesis methods*

CH_3_CN was refluxed over P_2_O_5_ for 2 h and then distilled in dry nitrogen, and K_2_CO_3_ was dried at 200 ℃ for 4 h. Pentaacetylquercetin was synthesized using pyridine/acetic anhydride and purified by repeated recrystallization in acetone. Step1: A mixture of pentaacetylquercetin 1.28 g, K_2_CO_3 _0.69 g, 18-crown-6 33 mg, CH_3_CN 50 mL and halogenated hydrocarbons (n-butyl bromide for BQ and DBQ, allyl chloride for AQ and DAQ, cinnamyl chloride for CQ and DCQ, geranyl bromide for GQ) was heated and gently stirred under dry nitrogen for 72 h. After filtration, the filtrate was extracted and washed by 10 mL petroleum ether for three times, followed by evaporation in rotate evaporator. Step 2: The residue was hydrolyzed by 30 mL boiling 10% NaOH/ethanol (1:2, v/v) in nitrogen for 10 min. After cooling in nitrogen, the hydrolyzed product was neutralized with 30 mL of 1 M HCl and then filtered. The filter cake was washed by water and then dried in vacuum at room temperature. The derivatives were purified as follow. The crude products were dissolved in 5 mL hot ethyl acetate and then 50 mL petroleum ether was added. After cooling, yellow powder was collected by filtering. The yellow powder obtained above were further purified using column chromatography on silica gel (300–400 mesh, Qingdao Ocean Chemical Company, Qingdao, China). A mixture of petroleum ether and ethyl acetate was used as mobile phase in this step. Monoalkyl and dialkyl derivatives were separated after this step. 

Compound BQ: yellow solid, overall yield 52% in three steps of reactions. Approximately 0.6 mL of n-butyl bromide was used in step 1. ^1^H NMR (400 MHz, DMSO-*d6*): δ 6.32 (d, 1H, J = 2.19 Hz, 6-H), 6.69 (d, 1H, J = 2.14 Hz, 8-H), 7.75 (d, 1H, J = 2.21 Hz, 2′-H), 6.9 (d, 1H, J = 8.48 Hz, 5′-H), 7.58 (dd, 1H, J = 8.48, 2.18 Hz, 6′-H), 9.45 (s, 1H, 3-OH), 12.5 (s, 1H, 5-OH), 9.27 (s, 1H, 3′-OH), 9.62 (s, 1H, 4′-OH), 4.11 (t, 2H, J = 6.52 Hz, 1′′-H), 1.72 (quint, 2H, J = 7.02 Hz, 2′′-H), 1.45 (sext, 2H, J = 7.46 Hz, 3′′-H), 0.94 (t, 3H, J = 7.38 Hz, 4′′-H); NOESY (400 MHz, DMSO-*d6*) demonstrated that there were two groups of related hydrogen: 6-H/1′′-H and 8-H/1′′-H; ^13^C NMR (400 MHz, DMSO-*d*6): δ 147.2 (2-C), 136.84 (3-C), 176.47 (4-C), 160.83 (4a-C), 98.22 (5-C), 164.85 (6-C), 92.73 (7-C), 156.58 (8-C), 104.41 (8a-C), 123.7 (1′-C), 115.32 (2′-C), 146.83 (3′-C), 149.28 (4′-C), 113.22 (5′-C), 120.22 (6′-C), 68.59 (1′′-C), 31.23 (2′′-C), 19.15 (3′′-C), 14.19 (4′′-C); ESI-MS: *m/z* 357 [M – H]^-^.

Compound DBQ: yellow solid, overall yield 21% in three steps of reactions. Approximately 0.6 mL of n-butyl bromide was used in step 1. ^1^H NMR (400 MHz, DMSO-*d6*): δ 6.31 (d, 1H, J = 2.11 Hz, 6-H), 6.68 (d, 1H, J = 2.11 Hz, 8-H), 7.75 (d, 1H, J = 2.19 Hz, 2′-H), 7.06 (d, 1H, J = 8.76 Hz, 5′-H), 7.65 (dd, 1H, J = 8.74, 2.17 Hz, 6′-H), 12.4 (s, 1H, 5-OH), 9.18 (s, 1H, 3′-OH), 9.53 (s, 1H, 4′-OH), 4.03-4.09 (m, 4H, 1′′-H, 1′′′-H), 1.70-1.72 (m, 4H, 2′′-H, 2′′′-H), 1.41-1.50 (m, 4H, 3′′-H, 3′′′-H), 0.92-0.97 (m, 6H, 4′′-H, 4′′′-H); NOESY (400 MHz, DMSO-*d6*) demonstrated that there were no more related hydrogen atoms than that of BQ; ^13^C NMR (400 MHz, DMSO-*d*6): 147.2 (2-C), 136.84 (3-C), 176.47 (4-C), 160.83 (4a-C), 98.22 (5-C), 164.85 (6-C), 92.73 (7-C), 156.58 (8-C), 104.41 (8a-C), 123.7 (1′-C), 115.32 (2′-C), 146.83 (3′-C), 149.28 (4′-C), 113.22 (5′-C), 120.22 (6′-C), 68.59 (1′′-C), 31.23 (2′′-C), 19.15 (3′′-C), 14.19 (4′′-C), 68.38 (1′′′-C), 30.92 (2′′′-C), 19.09 (3′′′-C), 14.07 (4′′′-C); ESI-MS: *m/z* 413 [M – H]^-^.

Compound AQ: yellow solid, overall yield 50% in three steps of reactions. Approximately 0.6 mL of allyl chloride was used in step 1. ^1^H NMR (400 MHz, DMSO-*d6*): δ 6.37 (d, 1H, J = 1.97 Hz, 6-H), 6.71 (d, 1H, J = 1.93 Hz, 8-H), 7.73 (d, 1H, J = 1.95 Hz, 2′-H), 6.9 (d, 1H, J = 8.53 Hz, 5′-H), 7.57 (dd, 1H, J = 8.53, 1.92 Hz, 6′-H), 9.43 (s, 1H, 3-OH), 12.5 (s, 1H, 5-OH), 9.33 (s, 1H, 3′-OH), 9.47 (s, 1H, 4′-OH), 4.71 (d, 2H, J = 5.14 Hz, 1′′-H), 6.02-6.11 (m, 1H, 2′′-H), 5.44 (dd, 1H, J = 17.32, 1.22 Hz, 3′′cis-H), 5.31 (dd, 1H, J = 10.59, 1.08 Hz, 3′′trans-H); NOESY (400 MHz, DMSO-*d6*) demonstrated that there were two groups of related hydrogen: 6-H/1′′-H and 8-H/1′′-H; ^13^C NMR (400 MHz, DMSO-*d*6): 147.78 (2-C), 136.49 (3-C), 176.39 (4-C), 160.86 (4a-C), 98.32 (5-C), 164.19 (6-C), 93.07 (7-C), 156.45 (8-C), 104.53 (8a-C), 122.33 (1′-C), 116.03 (2′-C), 145.54 (3′-C), 148.31 (4′-C), 115.73 (5′-C), 120.48 (6′-C), 69.36 (1′′-C), 133.36 (2′′-C), 118.53 (3′′-C); ESI-MS: *m/z* 341 [M – H]^-^.

Compound DAQ: yellow solid, overall yield 25% in three steps of reactions. Approximately 0.6 mL of allyl chloride was used in step 1. ^1^H NMR (400 MHz, DMSO-*d6*): δ 6.38 (d, 1H, J = 2.16 Hz, 6-H), 6.72 (d, 1H, J = 2.14 Hz, 8-H), 7.75 (d, 1H, J = 2.16 Hz, 2′-H), 7.1 (d, 1H, J = 8.72 Hz, 5′-H), 7.65 (dd, 1H, J = 8.69, 2.17 Hz, 6′-H), 12.45 (s, 1H, 5-OH), 9.33 (s, 1H, 3′-OH), 9.56 (s, 1H, 4′-OH), 4.71 (d, 2H, J = 5.23 Hz, 1′′-H), 4.65 (d, 2H, J = 5.2 Hz, 1′′′-H), 6.04-6.09 (m, 2H, 2′′-H, 2′′′-H), 5.42-5.49 (m, 2H, 3′′cis-H, 3′′′cis-H), 5.27-5.32 (m, 2H, 3′′trans-H, 3′′′trans-H); NOESY (400 MHz, DMSO-*d6*) demonstrated that there were no more related hydrogen atoms than that of AQ; ^13^C NMR (400 MHz, DMSO-*d*6): 147.21 (2-C), 136.95 (3-C), 176.54 (4-C), 160.88 (4a-C), 98.36 (5-C), 164.28 (6-C), 93.13 (7-C), 156.51 (8-C), 104.58 (8a-C), 124.02 (1′-C), 115.54 (2′-C), 146.93 (3′-C), 148.73 (4′-C), 113.82 (5′-C), 120.11 (6′-C), 69.38 (1′′, 1′′′-C), 133.36 (2′′-C), 118.54 (3′′-C), 134.05 (2′′′-C), 118.1 (3′′′-C); ESI-MS: *m/z* 381 [M – H]^-^.

Compound CQ: yellow solid, overall yield 31% in three steps of reactions. Approximately 0.7 mL of cinnamyl chloride was used in step 1. ^1^H NMR (400 MHz, DMSO-*d6*): δ 6.42 (d, 1H, J = 2.18 Hz, 6-H), 6.78-6.82 (m, 2H, 8-H,3′′-H), 7.75 (d, 1H, J = 2.14 Hz, 2′-H), 6.91 (d, 1H, J = 8.51 Hz, 5′-H), 7.6 (dd, 1H, J = 8.45, 2.16 Hz, 6′-H), 9.52 (s, 1H, 3-OH), 12.5 (s, 1H, 5-OH), 9.31 (s, 1H, 3′-OH), 9.69 (s, 1H, 4′-OH), 4.86 (d, 2H, J = 5.72 Hz, 1′′-H), 6.54 (dt, 1H, J = 15.99, 5.76 Hz, 2′′-H), 7.50-7.52 (m, 2H, Ar2′′-H, Ar6′′-H), 7.34-7.38 (m, 2H, Ar3′′-H,Ar5′′-H), 7.27-7.30 (m, 1H, Ar4′′-H); NOESY (400 MHz, DMSO-*d6*) demonstrated that there were two groups of related hydrogen: 6-H/1′′-H and 8-H/1′′-H; ^13^C NMR (400 MHz, DMSO-*d*6): 147.77 (2-C), 136.51 (3-C), 176.39 (4-C), 160.87 (4a-C), 98.36 (5-C), 164.27 (6-C), 93.1 (7-C), 156.46 (8-C), 104.54 (8a-C), 122.33 (1′-C), 116.03 (2′-C), 145.54 (3′-C), 148.31 (4′-C), 115.74 (5′-C), 120.48 (6′-C), 69.32 (1′′-C), 124.47 (2′′-C), 133.54 (3′′-C), 136.44 (Ar1′′-C), 127.01 (Ar2′′-C, Ar6′′-C), 129.16 (Ar3′′-C, Ar5′′-C), 128.5 (Ar4′′-C); ESI-MS: *m/z* 417[M – H]^-^.

Compound DCQ: yellow solid, overall yield 8% in three steps of reactions. Approximately 0.7 mL of cinnamyl chloride was used in step 1. ^1^H NMR (400 MHz, DMSO-*d6*): δ 6.43 (d, 1H, J = 2.13 Hz, 6-H), 6.79-6.86 (m, 3H, 8-H, 3′′-H, 3′′′-H), 7.78 (d, 1H, J = 2.09 Hz, 2′-H), 7.18 (d, 1H, J = 8.64 Hz, 5′-H), 7.68 (dd, 1H, J = 8.62, 2.17 Hz, 6′-H), 12.48 (s, 1H, 5-OH), 9.4 (s, 1H, 3′-OH), 9.62 (s, 1H, 4′-OH), 4.82-4.86 (m, 4H, 1′′-H, 1′′′-H), 6.52-6.58 (m, 2H, 2′′-H, 2′′′-H), 7.49-7.52 (m, 4H, Ar2′′-H, Ar6′′-H, Ar2′′′-H, Ar6′′′-H), 7.34-7.38 (m, 4H, Ar3′′-H, Ar5′′-H, Ar3′′′-H, Ar5′′′-H), 7.27-7.30 (m, 2H, Ar4′′-H, Ar4′′′-H); NOESY (400 MHz, DMSO-*d6*) demonstrated that there were no more related hydrogen atoms than that of CQ; ^13^C NMR (400 MHz, DMSO-*d*6): 147.21 (2-C), 136.64 (3-C), 176.54 (4-C), 160.9 (4a-C), 98.4 (5-C), 164.36 (6-C), 93.16 (7-C), 156.54 (8-C), 104.6 (8a-C), 124.06 (1′-C), 115.56 (2′-C), 146.97 (3′-C), 148.81 (4′-C), 113.89 (5′-C), 120.16 (6′-C), 69.34 (1′′-C), 124.44 (2′′-C), 133.57 (3′′-C), 136.44 (Ar1′′-C), 127.01 (Ar2′′-C, Ar6′′-C), 129.15 (Ar3′′-C, Ar5′′-C, Ar3′′′-C, Ar6′′′-C), 128.5 (Ar4′′-C), 69.28 (1′′′-C), 125.28 (2′′′-C), 133 (3′′′-C), 136.64 (Ar1′′′-C), 126.92 (Ar2′′′-C, Ar5′′′-C), 128.36 (Ar4′′′-C); ESI-MS: *m/z* 533 [M – H]^-^.

Compound GQ: yellow solid, overall yield 47% in three steps of reactions. Approximately 0.7 mL of geranyl bromide was used in step 1. ^1^H NMR (400 MHz, DMSO-*d6*): δ 6.33 (d, 1H, J = 2.12 Hz, 6-H), 6.71 (d, 1H, J = 2.15 Hz, 8-H), 7.76 (d, 1H, J = 2.17 Hz, 2′-H), 6.91 (d, 1H, J = 8.51 Hz, 5′-H), 7.57 (dd, 1H, J = 8.62, 2.15 Hz, 6′-H), 9.49 (s, 1H, 3-OH), 12.51 (s, 1H, 5-OH), 9.28 (s, 1H, 3′-OH), 9.69 (s, 1H, 4′-OH), 4.72 (d, 2H, J = 6.43 Hz, 1′′-H), 5.42 (t, 1H, J = 6.34 Hz, 2′′-H), 1.74 (s, 3H, 3a′′-H), 2.06-2.08 (m, 4H, 4′′-H, 5′′-H), 5.05-5.06 (m, 1H, 6′′-H), 1.56 (s, 3H, 7a′′-H), 1.62 (s, 3H, 8′′-H); NOESY (400 MHz, DMSO-*d6*) demonstrated that there were two groups of related hydrogen: 6-H/1′′-H and 8-H/1′′-H; ^13^C NMR (400 MHz, DMSO-*d*6): 147.66 (2-C), 136.47 (3-C), 176.36 (4-C), 160.76 (4a-C), 98.35 (5-C), 164.51 (6-C), 92.95 (7-C), 156.45 (8-C), 104.37 (8a-C), 124.17 (1′-C), 116 (2′-C), 145.52 (3′-C), 148.27 (4′-C), 115.71 (5′-C), 120.41 (6′-C), 65.76 (1′′-C), 122.35 (2′′-C), 141.57 (3′′-C), 16.83 (3a′′-C), 39.33 (4′′-C), 26.19 (5′′-C), 119.31 (6′′-C), 131.5 (7′′-C), 18 (7a′′-C), 25.87 (8′′-C); ESI-MS: *m/z* 437 [M – H]^-^.

*Solubility test*

Solubility of the derivatives was evaluated by turbidimetry as previously described (7). Briefly, derivatives were dissolved in DMSO at a final concentration of 50 mM, and then diluted by DMEM medium at final concentrations of 20, 40, 60, 80, 100, 120, 140, 160, 180 and 200 μM. After these solutions were incubated at 37 ℃ for 48 h, absorbances of them at 630 nm were measured. 

*Cytotoxicity test*

Cytotoxicity, which is represented by concentration that shows 50% inhibition in cell proliferation (IC_50_), was evaluated by modified MTT (3-(4,5-dimethylthiazol-2-yl)- 2,5-diphenyltetrazolium bromide) assay as described before (8, 9). Taking solubility of the derivatives into consideration, BQ and GQ were selected for the primary cytotoxicity study. According to the result of the primary cytotoxicity study, the cytotoxicity of GQ on NCI-H446, A549, MGC-803 and SGC-7901 cell lines was further investigated. To justify the effectiveness of BQ and GQ, cytotoxicity of the parent compound quercetin on all the cell lines used in this study was determined either. Briefly, 8000 cells with 0.1 mL medium were seeded in 96-well microplates. The derivatives or quercetin were dissolved in DMSO (50 mM) and then diluted with the medium to a series of testing concentrations. After exposure to the derivatives or quercetin for 48 h, cells were carefully washed by PBS three times and then 0.1 mL MTT (0.5 mg/mL) was added to each well. DMSO was used as solvent for following spectrum analysis at 570 nm. Proliferation values were calculated with the formula as follow: proliferation = (A_test_-A_blank_)/(A_control_-A_blank_). According to the proliferation data, all IC_50_ values were calculated by SPSS software using probit regression assay.

**Table 1:** Inhibitory effects of quercetin and its derivatives on MCF-7 and Caco-2 cells

**Compounds**	**IC**_50_ ** (** **μM** **)**	
	**MCF-7**	**Caco-2**
Quercetin	343	340
BQ	38.6	66.8
GQ	20.2	43.7

**Table 2:** Inhibitory effects of quercetin and GQ on NCI-H446, A549, MGC-803 and SGC-7901 cells

	IC_50_ (μM)			
	NCI-H446	A549	MGC-803	SGC-7901
Quercetin	68.9	77.2	80.6	75.7
GQ	27.6	29.5	25.4	18.5

**Scheme 1:**
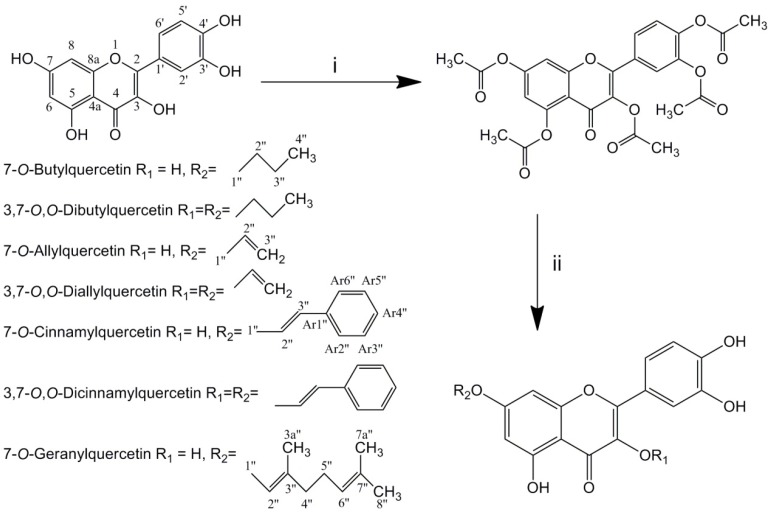
then 85 ℃, 10 min; (ii) halogenated hydrocarbons, K_2_CO_3_, 18-crown-6, dry CH_3_CN, 70 ℃, 72 h, then NaOH, EtOH, H_2_O, reflux, 10 min

**Figure 1:**
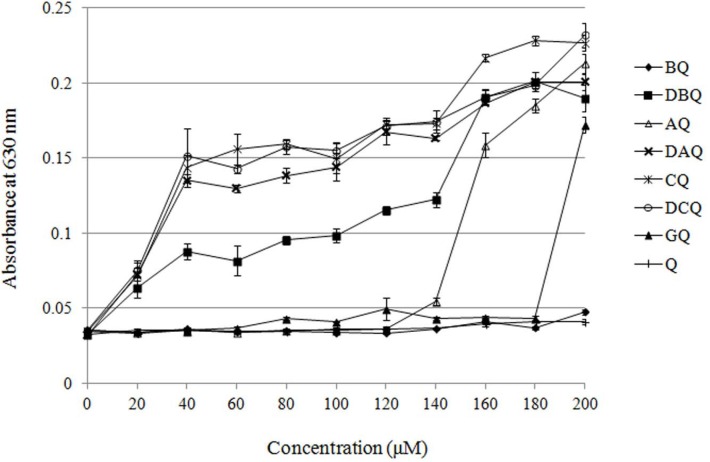
Solubility of the derivatives in DMEM medium evaluated by turbidimetry

## Results and Discussion

*Chemistry*

Total seven alkylated derivatives were synthesized via three steps: acetylation of quercetin in pyridine/acetic anhydride, alkylation of pentaacetylquercetin by halogenated hydrocarbons with K_2_CO_3_ and then deacetylation under alkaline condition (Scheme 1.). 7-*O*-monoalkyl and 3,7-*O*,*O*-dialkyl derivatives were obtained simultaneously in same reactions. Using more halogenated hydrocarbons in alkylating reaction would gain more 3,7-*O*,*O*-dialkyl derivatives. However, only trace 3,7-O,O-digeranylquercetin (DGQ) was obtained after synthesis, and it could not be isolated. 7-*O*-Butylquercetin (BQ), 7-*O*-allylquercetin (AQ), 7-*O*-cinnamylquercetin (CQ) and 7-*O*-geranylquercetin (GQ) were obtained as yellow amorphous powder, while 3,7-*O*,*O*-dibutylquercetin (DBQ), 3,7-*O*,*O*-diallylquercetin (DAQ) and 3,7-*O*,*O*-cinnamylquercetin (DCQ) were obtained as yellow needle-like crystal. Both of these derivatives and quercetin had similar UV spectrum (254, 370 nm).

*Solubility in DMEM medium*

To ensure the maximum concentration applied in cytotoxicity evaluation, solubility of these derivatives was measured by turbidimetry. As shown in Figure 1. all of the dialkyl derivatives and CQ were almost insoluble in DMEM medium, while BQ and GQ resulted in well solubility which was about 180 μM. The dialkyl derivatives and CQ might be too lipophilic to dissolve in the medium. Although the solubility of AQ was about 140 μM, its inhibition rate to MCF-7 cells was less than 50% at the highest concentration. The length of alkyl chains bears little relationship to the solubility of the derivatives. Taking their solubility into consideration, cytotoxicity of BQ and GQ was evaluated in this study. 

*Cytotoxicity*

Cytotoxicity of BQ, GQ and the parent compound quercetin against MCF-7 cells (human breast cancer cells) and Caco-2 cells (human colon cancer cells) was evaluated using MTT assay. As shown in Table 1. IC_50 _values of BQ and GQ to MCF-7 and Caco-2 cells lines were much lower than those of quercetin. BQ and GQ demonstrated strong cytotoxicity to the two cell lines, and GQ was the most toxic derivative. For 7-*O*-monoalkyl derivatives, a longer substituent group might have higher cytotoxicity. This phenomenon might be relative to lipophilicity and membrane penetration ability of the compounds.

Cytotoxicity of GQ to more human cancer cell lines was further evaluated and compared with that of quercetin. The cell lines included NCI-H446 (human lung cancer cells), A549 (human lung cancer cells), MGC-803 (human gastric cancer cells) and SGC-7901 (human gastric cancer cells). As shown in Table 2. GQ exhibited strong cytotoxicity against all of these four cancer cell lines and the cytotoxicity of GQ was stronger than that of quercetin. Consequently, GQ is a broad spectrum cytotoxic agent and it may be a promising leading drug for chemotherapy.

Previous studies demonstrated that quercetin and some of its derivatives could induce apoptosis in cancer cells via different pathways (10-12), and they could reverse multidrug resistance of tumor cells simultaneously (13, 14). The cytotoxicity of alkylated quercetin derivatives is strong according to our data, and further studies on apoptotic mechanism and multidrug resistance reversing are essential and meaningful. 

## Conclusions

In summary, seven alkylated quercetin derivatives were designed and synthesized. All of the dialkyl derivatives and the monoalkyl derivative CQ have poor solubility, while that of the monoalkyl derivatives BQ and GQ is better. BQ showed moderate cytotoxicity against MCF-7 cells and Caco-2 cells, whereas GQ showed strong cytotoxicity against different kinds of cancer cells. Among these seven derivatives, GQ is the most suitable candidate drug for cancer treatment. Studies on the antitumor mechanism and multidrug resistance reversing effect of this compound are being conducted. 
